# Energy Drinks: Effects on Blood Pressure and Heart Rate in Children and Teenagers. A Randomized Trial

**DOI:** 10.3389/fcvm.2022.862041

**Published:** 2022-03-21

**Authors:** Felix Sebastian Oberhoffer, Pengzhu Li, André Jakob, Robert Dalla-Pozza, Nikolaus Alexander Haas, Guido Mandilaras

**Affiliations:** Division of Pediatric Cardiology and Intensive Care, University Hospital, LMU Munich, Munich, Germany

**Keywords:** Energy Drinks, blood pressure, heart rate, pediatrics, prevention

## Abstract

**Background:**

The consumption of Energy Drinks (ED) is very popular among children and teenagers. While potential cardiovascular side effects of these beverages are suggested, the acute impact of ED consumption on the pediatric cardiovascular system has not been systematically examined yet. The aim of this study was to investigate the acute effects of ED consumption on blood pressure and heart rate in healthy children and teenagers.

**Methods:**

This study was a randomized, single-blind, placebo-controlled, crossover clinical trial. On two consecutive days, the study participants were asked to consume a weight-adjusted amount of an ED (3 mg caffeine per kg of body weight) or a placebo containing a similar amount of sugar but without conventional ED ingredients. Systolic blood pressure (SBP), diastolic blood pressure (DBP) and heart rate were measured at the following time points: baseline as well as 30, 60, 120 and 240 min after beverage consumption.

**Results:**

In total, 27 healthy children and adolescents (mean age 14.53 ± 2.40 years, 14 male) were included in the present study. Compared to placebo intake, mean SBP was demonstrated to be up to 5.23 mmHg (*p* < 0.0001) and mean DBP up to 3.29 mmHg (*p* < 0.001) increased after ED consumption. Prevalence of elevated blood pressure, stage 1 and stage 2 hypertension was higher after ED consumption. Heart rate tended to be lower after ED consumption.

**Conclusions:**

The acute ED consumption is associated with a significantly increased SBP and DBP in healthy children and teenagers. Minors, particularly those with pre-existing health conditions, should be discouraged from drinking EDs.

**Clinical Trial Registration:**

https://www.drks.de/drks_web/, identifier: DRKS00027580.

## Introduction

Energy drinks (ED) are sweetened beverages, which contain high amounts of caffeine and other stimulants, such as taurine, guarana or glucuronolactone. In recent years, EDs have become more and more popular, particularly with children and teenagers.

According to a survey conducted by the European Food Safety Authority, two thirds of European adolescents between 10 and 18 years of age consume EDs (1). In correlation with their popularity, reports of emergency department visits linked to ED consumption are rising: The excessive consumption of EDs, especially in combination with party drugs or in the presence of a cardiac condition, is associated with adverse cardiovascular events (e.g. ventricular arrhythmia, supraventricular arrhythmia, myocardial ischemia) (2). Interestingly, a significantly prolonged QTc interval in adult study subjects was demonstrated in several clinical trials after ED consumption (3, 4). This phenomenon might partially explain the increased occurrence of rhythm disorders seen after excessive ED consumption (4). In addition to the arrhythmogenic potential of EDs, studies examining adult subjects were able to reveal a significant increase in blood pressure after ED consumption (3–5).

Medical associations, including the American Academy of Pediatrics and the American Medical Association, advise against ED consumption in minors (6, 7). Although children and teenagers represent one of the main ED consumer groups (1), the acute effects of ED consumption on the pediatric cardiovascular system have not been evaluated yet.

The aim of this study was to investigate the acute effects of ED consumption on blood pressure and heart rate in healthy children and teenagers by conducting a randomized, single-blind, placebo-controlled, crossover clinical trial.

## Materials and Methods

### Ethical Statement

The Ethics Committee of the Ludwig Maximilians University Munich (Munich, Germany) approved this study on January 12th, 2021 (project number 20-0993). Prior written informed consent was obtained from all study participants and in underage study participants additionally from parents or legal guardians.

### Patient and Public Involvement Statement

Patients or the public were not involved in the design, or conduct, or reporting, or dissemination plans of our research.

### Study Population

Healthy children and teenagers between the ages of 10 to 18 years were prospectively recruited for this study. Study participants were examined for eligibility before enrollment through a personal interview, clinical examination, conventional echocardiography, 24-h Holter ECG and 24-h blood pressure measurement. The following exclusion criteria were applied for potential study participants: presence of chronic conditions (e.g., congenital heart disease, arterial hypertension, presence of severe dysrhythmia), history of sudden heart death within the family, known allergies against beverage ingredients, regular use of medication with effects on the cardiovascular function, regular use of drugs including smoking and alcohol consumption, pregnancy.

In study participants <18 years of age, weight classification was assessed according to body mass index (BMI, kg/m^2^) percentiles (P.) established by Kromeyer-Hauschild et al. (normal weight if BMI <90. P., overweight if BMI ≥ 90. P. but <97. P., obese if BMI ≥ 97. P.) (8). In study participants ≥ 18 years of age, normal weight was defined as BMI <25 kg/m^2^, overweight as BMI ≥ 25 kg/m^2^ but <30 kg/m^2^ and obesity as BMI ≥ 30 kg/m^2^.

General caffeine consumption behavior of study participants was evaluated in accordance with Shah et al.: Rare caffeine consumer if <1 caffeine containing drink per month, occasional caffeine consumer if 1 to 3 caffeine containing drinks per month, frequent caffeine consumers if 1 to 6 caffeine containing drinks per week and daily caffeine consumers if ≥ 1 caffeine containing drink per day (4). Moreover, ED consumption behavior of study participants was investigated as specified above.

### Study Design

This study was a randomized, single-blind (study participants), placebo-controlled, crossover clinical trial conducted between April 2021 to October 2021 by the Division of Pediatric Cardiology and Intensive Care, University Hospital, LMU Munich (Munich, Germany). The study was registered in the German Clinical Trials Register (https://www.drks.de/drks_web/, DRKS00027580.) The study design was adapted to a prior study performed in adult subjects by Shah et al. (4).

Eligible study participants were required not to consume any sources of caffeine (e.g., coffee, tea, chocolate) or drugs (e.g., tobacco, alcohol) 48 h before and 24 h after study participation. An overnight fast (with allowance for water) was asked preceding every study day. Study participants were requested not to consume any food or liquids during each day's study duration.

Study participants were randomized into one of two intervention phases by coin flipping. On two consecutive days, the study participants received a commercially available caffeinated ED or a placebo drink without conventional ingredients found in an ED (e.g., caffeine, taurine). The amount of administered ED was bodyweight-adjusted (3 mg caffeine per kilogram of bodyweight) and represented the acute maximal caffeine consumption for healthy children and teenagers considered as safe by the European Food Safety Authority (9). The amount of administered placebo-drink was matched to the ED. According to the ingredient label, the ED contained caffeine (32 mg/100 mL), taurine (200 mg/100 mL), glucuronolactone (24 mg/10 mL), ginseng aroma extract (10 mg/100 mL), guarana extract (10 mg/100 mL) and vitamins. The placebo drink contained, according to the ingredient label, carbonated water, multi-fruit juice as well as fruit and vegetable extracts. Both beverages were similar in sugar content (ED: 15,2 g/100 mL, placebo drink: 13,2 g/100 mL) and taste. Both beverages were administered in an identical and masked drinking bottle at room temperature on each study day.

Blinding quality was assessed as follows: After complete data collection, study participants were asked to guess on what study day the ED beverage was administered.

### End Point Measurement

The primary end points were systolic blood pressure (SBP, mmHg), diastolic blood pressure (DBP, mmHg) and heart rate (HR, bpm). End points were evaluated at baseline as well as 30, 60, 120 and 240 min after beverage consumption on each study day. On both study days, beverages were administered at similar morning hours to minimize circadian rhythm changes (4). In addition, study participants were provided a sickbed for each study day and requested to stay in the supine position for the whole study duration to reduce the potential influence of physical activity on the cardiovascular parameters studied.

Brachial blood pressure measurement was performed in a supine position utilizing an automated blood pressure device (Infinity M540, Dräger, Germany). In study participants <16 years of age, the presence of arterial hypertension was assessed according to blood pressure percentiles (P.) established by Neuhauser et al. (normal if blood pressure <90. P.; elevated if blood pressure ≥ 90. P but <95. P.; stage 1 hypertension if blood pressure ≥ 95. P but <99. P. + 5 mmHg and stage 2 hypertension if blood pressure ≥ 99. P. + 5 mmHg) (10). In study participants ≥ 16 years of age, the presence of arterial hypertension was defined in accordance with the “2017 ACC/AHA Guideline for the Prevention, Detection, Evaluation, and Management of High Blood Pressure in Adults” (11).

Conventional 12-lead ECGs were recorded in supine positions. HR was automatically calculated by the ECG-devices (MAC 5500 / CardioSoft V6.73, General Electrics Healthcare, USA) and used for further analysis.

### Statistical Analysis

This study was a pediatric pilot study. To the best of our knowledge, pediatric reference values of change in blood pressure and HR after ED consumption do not exist and can thus not be considered in a power analysis. A maximum baseline-corrected, placebo-adjusted change of + 4.6 mmHg (SD ± 5 mmHg, 80% power, α = 5%) in SBP by the ED was assumed according to results demonstrated in an adult cohort by Shah et al. (4). The sample size test for matched samples and continuous outcome revealed that a minimum of 10 study participants was needed for final data analysis. Supposing a dropout rate of 25%, a minimum of 14 study participants had to be recruited for the present study. A two-way repeated-measures ANOVA was performed to evaluate the effect of different beverages on SBP, DBP, and HR over time (R version 4.1.1). Ln or Sqrt data transformation was used if data was not normally distributed. The Bonferroni adjusted pairwise test was used for *post-hoc* testing. The maximum change from baseline within the time frame was calculated and analyzed using the paired *t*-test. Data analysis was independently performed by a masked researcher. A *p*-value <0.05 was considered statistically significant.

## Results

In total, 27 healthy children and teenagers were included in the analysis. Study participants' characteristics are summarized in Table 1. None of the participants had any pre-existing health conditions or were taking medications. Cardiovascular parameters did not show significant differences between both groups at baseline (Table 2). Thirteen out of 27 study participants (48.15%) correctly guessed the day of ED administration, suggesting appropriate blinding quality.

**Table 1:** Study participants' characteristics (*n* = 27).

**Characteristics**	**Total**
Age, years (mean ± SD)	14.53 ± 2.40
Sex, n (%)	
Male	14 (51.85)
Female	13 (48.15)
Weight Classification, n (%)	
Normal weight	23 (85.19)
Overweight	4 (14.81)
Obese	0 (0)
Caffeine Consumption Behavior, n (%)^a^	
Rare	17 (62.96)
Occasional	3 (11.11)
Frequent	5 (18.52)
Daily	2 (7.41)
Energy Drink Consumption Behavior, n (%)^b^	
Never	12 (44.44)
Rare	11 (40.74)
Occasional	1 (3.70)
Frequent	3 (11.11)
Daily	0 (0)

a *Rare caffeine consumer if <1 caffeine containing drink per month, occasional caffeine consumer if 1 to 3 caffeine containing drinks per month, frequent caffeine consumer if 1 to 6 caffeine containing drinks per week and daily caffeine consumer if ≥ 1 caffeine containing drink per day (4)*.

b *Rare Energy Drink (ED) consumer if <1 ED per month, occasional ED consumer if 1 to 3 EDs per month, frequent ED consumer if 1 to 6 EDs per week and daily ED consumer if ≥ 1 ED per day*.

**Table 2:** Cardiovascular parameters at baseline (*n* = 27).

**Parameters**	**Energy Drink**	**Placebo**	***p*-value**
SBP (mmHg)	112.74 ± 8.67	113.15 ± 7.93	0.775
DBP (mmHg)	65.07 ± 8.43	65.26 ± 7.11	0.907
HR (bpm)	69.22 ± 11.29	70.89 ± 14.22	0.229

*Mean ± standard deviation is used for normally distributed variables. SBP, systolic blood pressure; DBP, diastolic blood pressure; HR, heart rate*.

### Acute Effects of Energy Drinks on Systolic Blood Pressure

**Figure 1:**
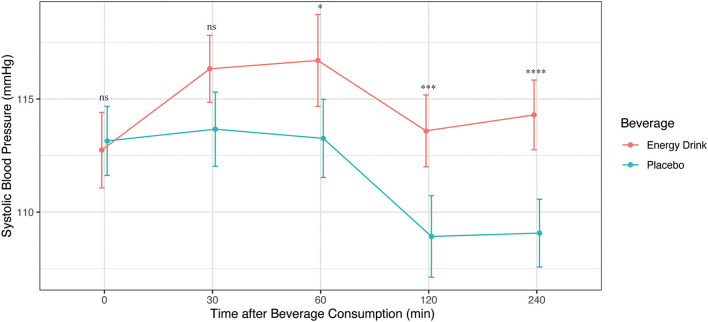
Systolic blood pressure (mmHg) after Energy Drink and placebo consumption at different time points *p < 0.05, ***p < 0.001, ****p < 0.0001.

The Shapiro-Wilk test revealed non-normal distribution for SBP at time 60 within the ED group. To achieve normal distribution, the original SBP data was transferred into Ln-form. According to Mauchly's spherical hypothesis test for the interaction term “beverage and time,” the variance and covariance matrices of the dependent variables were equal (*p* > 0.05). The interaction between the variables “beverage and time” had a statistically significant effect on SBP (*p* = 0.049). Hence, the separate effect of “beverage consumption” on SBP was additionally analyzed at each time point. At time 0 and at time 30 no significant difference in SBP was assessed after ED and placebo consumption. At time 60, time 120 and time 240, SBP was demonstrated to be significantly higher after ED consumption compared to placebo intake with a mean difference of 3.44 mmHg, 4.66 mmHg and 5.23 mmHg respectively. The separate effect of “beverage and time” on SBP is summarized in Table 3; Figure 1.

**Table 3:** The separate effect of beverage and time on systolic blood pressure (*n* = 27).

**Parameters**	**Energy Drink (mmHg)**	**Placebo (mmHg)**	***p*-value**
Time 0	112.74 ± 8.67	113.15 ± 7.93	0.776
Time 30	116.33 ± 7.69	113.67 ± 8.52	0.139
Time 60	116.70 ± 10.55	113.26 ± 8.97	0.045^*^
Time 120	113.59 ± 8.25	108.93 ± 9.36	0.000479^***^
Time 240	114.30 ± 8.01	109.07 ± 7.79	0.0000711^****^

*Mean ± standard deviation is used for normally distributed variables*.

* *p < 0.05*,

*** *p < 0.001*,

**** *p < 0.0001*.

**Figure 2:**
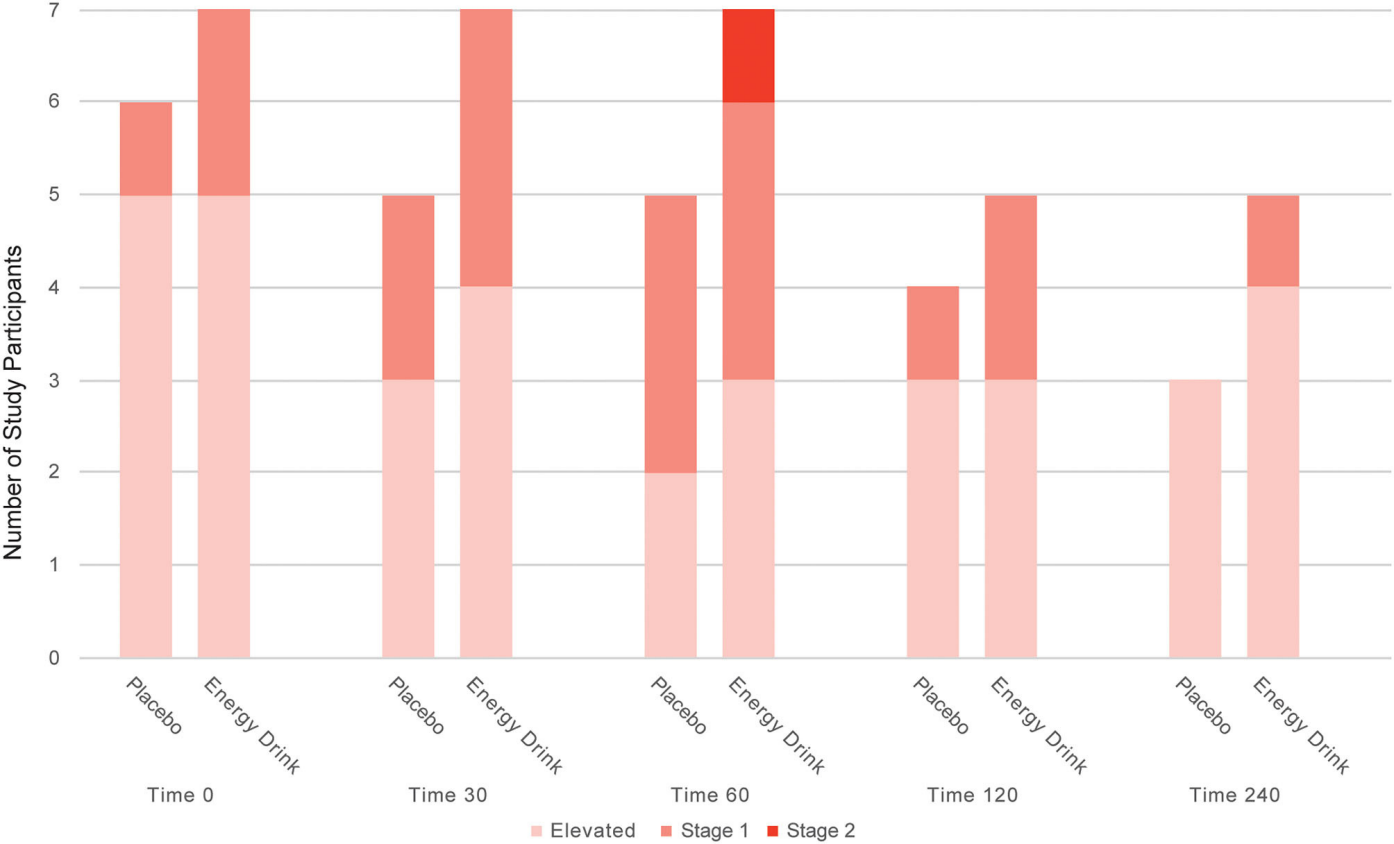
Number of study participants with elevated systolic blood pressure, stage 1 and stage 2 systolic hypertension after Energy Drink and placebo consumption at different time points.

The maximum change in SBP from baseline was significantly greater after ED consumption compared to placebo intake (5.41 ± 11.48 mmHg vs. −2.07 ± 12.53 mmHg, *p* = 0.022). In addition, the prevalence of elevated SBP as well as stage 1 and stage 2 systolic hypertension tended to be higher after ED consumption, compared to placebo intake within the study participants (Figure 2).

### Acute Effects of Energy Drinks on Diastolic Blood Pressure

**Figure 3:**
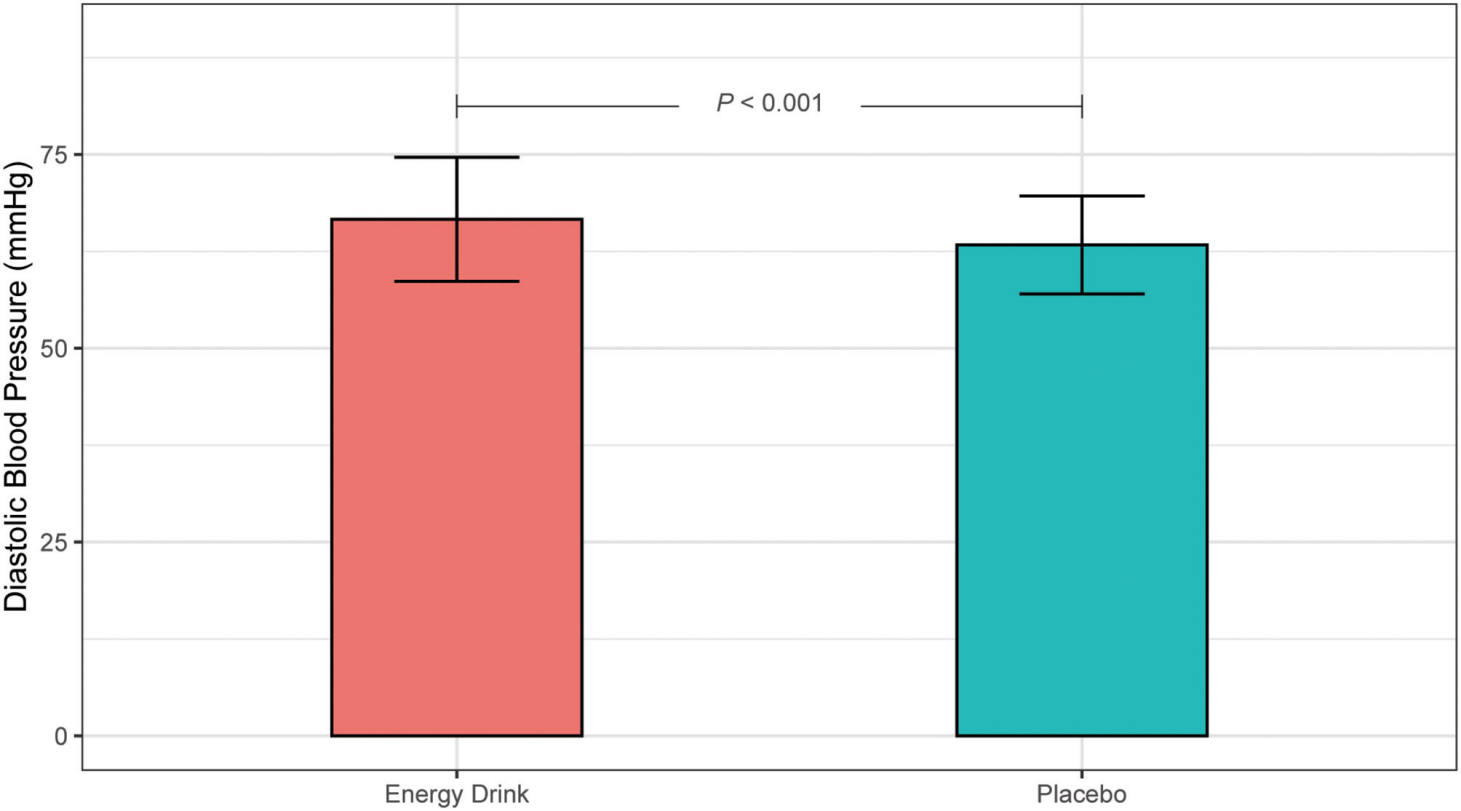
Main effect of Energy Drink and placebo consumption on diastolic blood pressure (mmHg).

The Shapiro-Wilk test revealed non-normal distribution for DBP at time 30 for both beverage groups. To achieve normal-distribution, the original DBP data was transformed into sqrt-form. According to Mauchly's spherical hypothesis test for the interaction term “beverage and time,” the variance and covariance matrices of the dependent variables were equal (*p* > 0.05). No significant interaction between “beverage and time” on DBP was demonstrated (*p* = 0.116). The main effect of the variable “beverage” on DBP was statistically significant (*p* < 0.001) and revealed a difference of 3.29 mmHg after ED and placebo consumption (66.64 ± 8.00 mmHg vs. 63.35 ± 6.33 mmHg, *p* < 0.001) (Figure 3).

**Figure 4:**
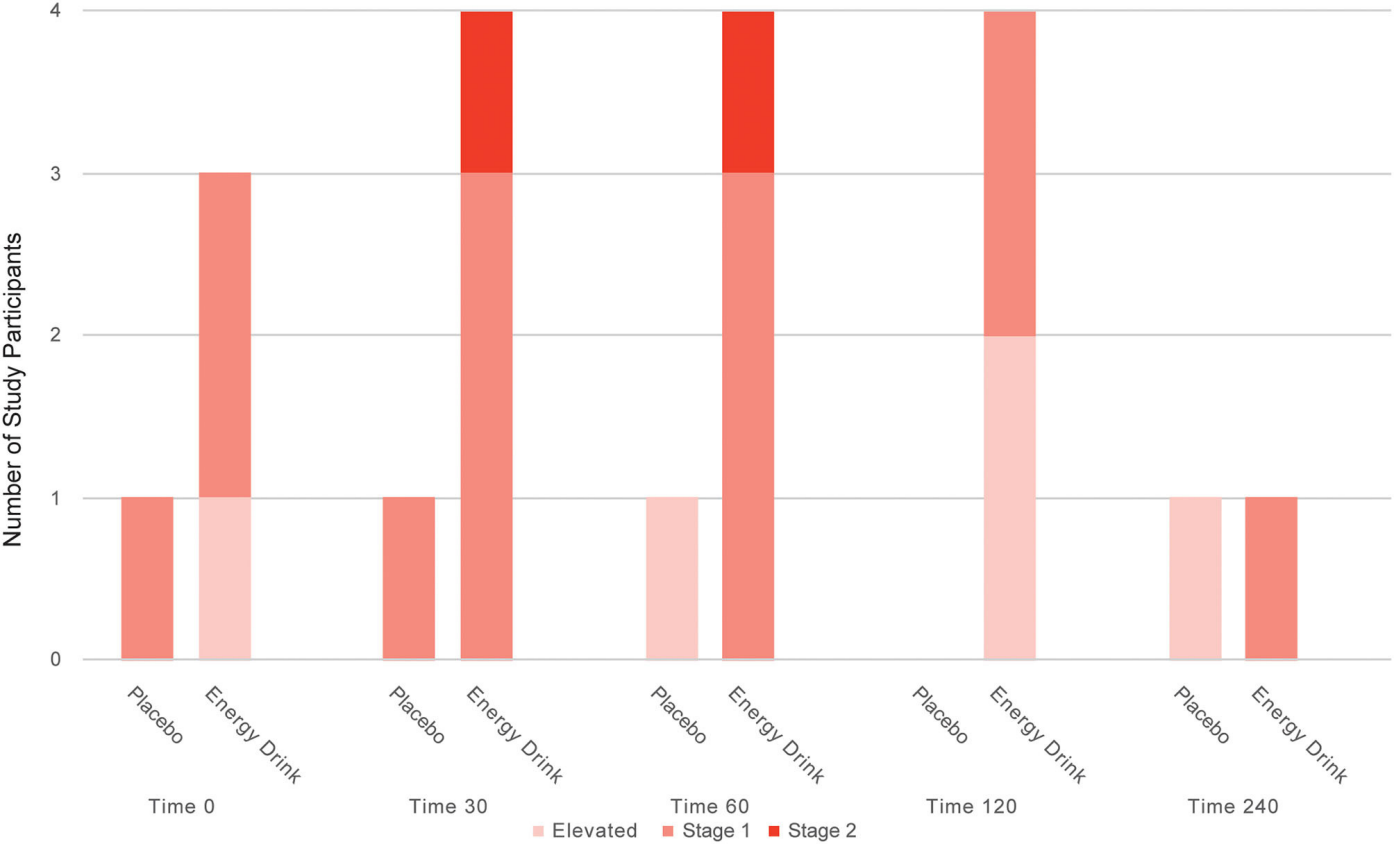
Number of study participants with elevated diastolic blood pressure, stage 1 and stage 2 systolic hypertension after Energy Drink and placebo consumption at different time points.

Moreover, the prevalence of elevated DBP as well as stage 1 and stage 2 diastolic hypertension tended to be higher after ED consumption, compared to placebo intake within the study participants (Figure 4).

The maximum change in DBP from baseline did, however, not differ significantly after ED consumption compared to placebo intake (2.81 ± 14.32 mmHg vs. −2.44 ± 11.72 mmHg, *p* = 0.181).

### Acute Effects of Energy Drinks on Heart Rate

The interaction between the variables “beverage and time” had no statistically significant effect on HR (*p* = 0.422). The main effect of the variable “beverage” on HR was not significant. However, HR tended to be lower after ED consumption compared to placebo intake (68.07 ± 11.25 bpm vs. 69.73 ± 11.58 bpm, *p* = 0.055). The maximum change in HR from baseline did not differ significantly after ED consumption compared to placebo intake (−2.44 ± 11.10 bpm vs. −0.15 ± 15.03 bpm, *p* = 0.428).

## Discussion

This is the first study investigating the acute effects of ED consumption on blood pressure and HR in healthy children and teenagers. A randomized, single-blind (study participants), placebo-controlled, crossover study design was applied to maximize data validity. In total, 27 healthy children and teenagers with a mean age of 14.53 years were included in the present study.

### Energy Drinks and Their Effect on Blood Pressure and Heart Rate: Physiological Considerations

In this study we were able to demonstrate a significant temporary elevation of SBP and DBP in healthy children and teenagers after ED consumption. Compared to placebo intake, mean SBP was demonstrated to be increased up to 5.23 mmHg and mean DBP up to 3.29 mmHg after ED consumption. Within adults, a meta-analysis by Shah et al. revealed an average increase of 4.44 mmHg for SBP and 2.73 mmHg for DBP after ED consumption (5). Therefore, the results of this study suggest, that the pediatric cardiovascular system might react even more severely to the ingredients found in EDs. In line with other studies, HR was not significantly influenced by beverage consumption but tended to be lower after ED intake (5). A recent publication of our department investigated the acute effects of ED consumption on heart rhythm and electrocardiographic time intervals in 26 of the present 27 study participants (12): By constantly monitoring HR through a portable 3-lead Holter ECG device, a significantly lower HR of 2.71 bpm was demonstrated during the time period of 60–120 min after ED consumption compared to the placebo intake (12).

**Figure 5:**
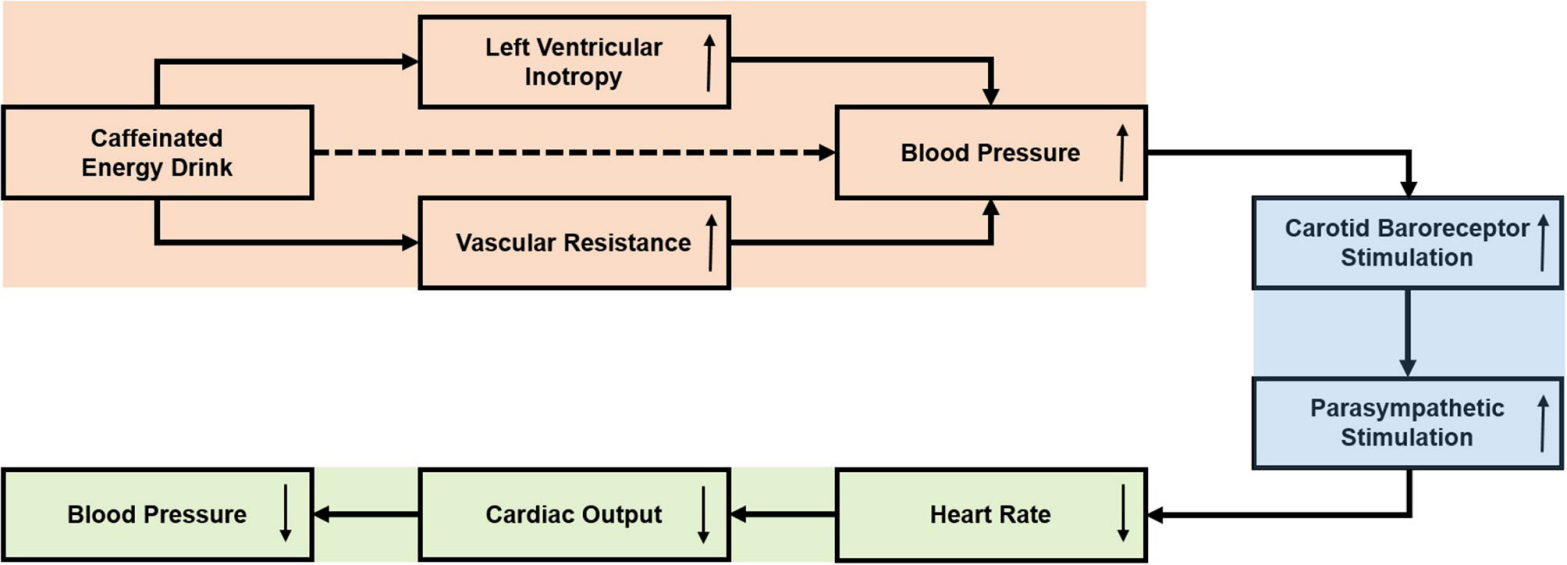
Effects of caffeinated Energy Drinks on cardiovascular function: physiological considerations.

The ED induced effects on the pediatric cardiovascular system can partially be explained by the great amount of caffeine and its derivates (guarana) found in such beverages (13). Caffeine is considered to increase left ventricular inotropy, act vasoconstrictive and thus elevate blood pressure (13). The caffeine induced elevation in blood pressure may lead to carotid baroreceptor activation and consequently to parasympathetic stimulation (14). The parasympathetic stimulation causes a decrease in HR. cardiac output and blood pressure (14). These physiological considerations (Figure 5) can potentially account for the results shown in this study: Peak caffeine plasma concentration is reached approximately 30 min after oral administration (15). In this study, an initial increase in SBP was assessed one hour after ED consumption. HR, in contrast, tended to be lower after ED consumption. Two and four hours after ED consumption, a decrease in SBP was demonstrated compared to peak SBP, potentially due to increased parasympathetic activity. To further evaluate these physiological considerations of caffeinated EDs on the cardiovascular system, studies are required that simultaneously monitor parasympathetic activation and electrocardiographic time intervals.

Besides caffeine, EDs contain other ingredients, such as taurine, glucuronolactone and B-vitamins. While the aminoacidic taurine is assumed to lower blood pressure (16, 17), the acute effects of glucuronolactone and B-vitamins on the cardiovascular system still need to be further studied.

### Energy Drinks: A Potential Health Threat for Minors?

According to the European Food Safety Agency, the consumption of up to 3 mg of caffeine per kilogram of bodyweight per day can be considered as safe for minors (9). In this study, the ED consumption of a comparable amount of caffeine led, compared to the placebo intake, to a significant increase in SBP and DBP with a mean difference of up to 5.23 and 3.29 mmHg respectively. In addition, children and teenagers presented more frequently with elevated blood pressure, stage 1 and stage 2 hypertension after ED consumption. Regarding the results demonstrated in this study, the upper caffeine limit for children and adolescents suggested by the European Food Safety Agency should be critically discussed and further research should be performed (9).

Children and teenagers are the main consumer group of ED products. According to a survey conducted by the European Food Safety Authority, two thirds of European adolescents between 10 and 18 years of age consume EDs (1). Twelve percent of these adolescent ED consumers are classified as “high chronic” consumers, drinking EDs at least four to five times per week (1). In addition, 12% percent of adolescent ED consumers drink at least 1.065 liters of ED in one setting and are therefore identified as “high acute” consumers (1). The high prevalence of chronic and excessive ED consumers among children and teenagers is alarming: While the chronic ED consumption increases the risk of arterial hypertension, glucose metabolism disorders and overweight, the excessive ED consumption, predominantly in combination with drugs, is associated with rhythm disorders (2). Interestingly, a recent study of our department revealed a significantly increased number of supraventricular extrasystoles after acute ED consumption (3 mg caffeine per kilogram of bodyweight) in 26 healthy children and teenagers (12). In addition, pediatric case reports suggest that the excessive ED consumption can potentially lead to acute renal failure (18), seizure (19) and spontaneous coronary artery dissection (20).

Particularly children and teenagers with pre-existing health conditions (e.g., arterial hypertension, rhythm anomalies, diabetes mellitus, excess weight) should be discouraged from drinking EDs.

### Strengths and Limitations

This is the first pediatric study investigating the acute effects of ED consumption on the cardiovascular system. A randomized, single-blind (study participants), placebo-controlled, crossover study design was applied to maximize data validity. In total, 27 healthy children and teenagers were included in the present study, exceeding distinctly the calculated sample size of 10 study participants. The single-blind (study participants) study design potentially could have led to some bias. Nonetheless, blood pressure and HR data collection was performed semi-automatically. Moreover, data analysis was conducted blinded. Special care was taken, to minimize beverage identification by study participants. Although ED and placebo used in this study were similar in taste and were administered in an identical and masked drinking bottle, some study participants potentially identified the administered beverage by taste, smell, or physical response. However, we consider the blinding quality to be appropriate, as only 48.15% of study participants correctly guessed the day of ED administration. For this study one specific ED product was used. The pediatric cardiovascular system might respond differentially to larger ED amounts, other ED products and to the combination of EDs with drugs (e.g., alcohol). As an overnight fast was required for each study day, the ED induced cardiovascular effects shown in this study might be overestimated. Further studies need to investigate ED induced cardiovascular effects without fasting conditions. Over 60% of study participants were rare caffeine consumers and over 40% have never drunk an ED. To minimize potential habitual caffeine effects, study participants were further required not to consume any sources of caffeine 48 h prior to study examination. However, the cardiovascular response after acute ED ingestion might potentially be lower in minors with habitual caffeine consumption. In this study, overweight subjects were included. The ED amount, however, was only matched to bodyweight and not to lean body mass. Overweight is associated with a higher body fat percentage. Therefore, the cardiovascular response of overweight participants might have been more severe due to higher amounts of caffeine per kg of lean body mass. Moreover, solely healthy children and teenagers were included in the present study. Minors with cardiovascular conditions (e.g., arterial hypertension, rhythm anomalies) might respond differently to the acute ED consumption. In addition, this study only assessed the acute cardiovascular effects of ED consumption. Hence, the impact of chronic ED consumption on the pediatric cardiovascular system remains uncertain and requires further research.

## Conclusions

The acute ED consumption is associated with a significantly increased SBP and DBP in healthy children and teenagers. In addition, the acute ED consumption tended to be associated with a decrease in HR. The excessive and chronic ED consumption under adolescents might even stronger influence the measured study variables and negatively affect the pediatric cardiovascular system, which requires further research. Particularly children and teenagers with pre-existing health conditions should be discouraged from drinking EDs.

## Data Availability Statement

The original contributions presented in the study are included in the article/supplementary material, further inquiries can be directed to the corresponding author.

## Ethics Statement

The Ethics Committee of the Ludwig Maximilians University Munich (Munich, Germany) approved this study on January 12th, 2021 (project number 20-0993). Prior written informed consent was obtained from all study participants and in underage study participants additionally from parents or legal guardians.

## Author Contributions

RDP, NH, FSO, and GM contributed to the conception, design, methodology, and provided administrative support. PL and FSO contributed to the formal analysis. RDP, NH, AJ, FSO, and GM provided the supervision support. All authors contributed to the investigation, data curation, validation, and original draft preparation. All authors contributed to manuscript revision, read, and approved the submitted version.

## Funding

This study (project title: EDUCATE-Study: Energy-Drinks – Unexplored Cardiovascular Alterations in TEens and TwEens) was supported by the German Heart Foundation/German Foundation of Heart Research.

## Conflict of Interest

The authors declare that the research was conducted in the absence of any commercial or financial relationships that could be construed as a potential conflict of interest.

## Publisher's Note

All claims expressed in this article are solely those of the authors and do not necessarily represent those of their affiliated organizations, or those of the publisher, the editors and the reviewers. Any product that may be evaluated in this article, or claim that may be made by its manufacturer, is not guaranteed or endorsed by the publisher.
